# Ethnic sensitivity analyses of pharmacokinetics, efficacy and safety in polycythemia vera treatment with ropeginterferon alfa-2b

**DOI:** 10.3389/fphar.2024.1455979

**Published:** 2024-09-24

**Authors:** Albert Qin, Daoxiang Wu, Jason Liao, Shuping Xie, Haoqi Chen, Yucheng Gao, Jie Cui, Xia Su, Narihisa Miyachi, Toshiaki Sato, Yaning Li, Jingjing Zhang, Weihong Shen, Wei Wang

**Affiliations:** ^1^ PharmaEssentia Corporation, Taipei, Taiwan; ^2^ PharmaEssentia Biotech (Beijing) Limited, Beijing, China; ^3^ Pharmaron Clinical Services Co., Ltd., Chengdu, China; ^4^ PharmaEssentia Japan KK, Tokyo, Japan

**Keywords:** ropeginterferon alfa-2b, ethnic sensitivity analyses, polycythemia vera, pharmacokinetics, safety

## Abstract

Ropeginterferon alfa-2b (Ropeg) is approved for the treatment of adults with polycythemia vera (PV). This report aims to analyze the ethnic sensitivity of Ropeg for the treatment of PV, comparing the pharmacokinetics (PK), efficacy, and safety profiles across diverse ethnic groups. We conducted a relevant review of PV and analysis of data obtained from clinical studies involving Ropeg. The PK behavior of ropeg showed no significant differences between Chinese and overseas populations. Their efficacy and safety profiles were similar across the ethnic groups. The analyses indicated that the dose-exposure-response profile of Ropeg was consistent irrespective of ethnic variations. The results suggest that Ropeg exhibits a consistent PK and pharmacodynamics profile and a similar therapeutic effect across different ethnic groups, confirming its efficacy and safety in the global treatment of PV. More generally, these findings support the broader application of Ropeg in diverse patient populations and emphasize the need for an inclusive clinical practice.

## 1 Introduction

Polycythemia vera (PV) is a common type of Philadelphia chromosome-negative myeloproliferative neoplasms (MPNs), which also include essential thrombocythemia (ET) and myelofibrosis (MF) (Guglielmelli and Vannucchi, 2020). It is characterized by the clonal proliferation of hematopoietic stem or progenitor cells, leading to the overproduction of red blood cells often accompanied by increased white blood cell (WBC) and platelet counts (Benevolo et al., 2023; Tefferi and Barbui, 2023). In the majority of cases, PV is associated with a gain-of-function mutation in the gene encoding Janus Kinase 2 (*JAK2*), termed *JAK2*V617F (Baxter et al., 2005; James et al., 2005; Levine et al., 2005; Kralovics et al., 2005). This mutation leads to the constitutive activation of the JAK-signal transducer and activator of transcription (STAT) signaling pathway, leading to the uncontrolled hematopoietic cell proliferation and increased blood counts (Chen and Mullally, 2014; Aruch and Mascarenhas, 2016).

Patients with PV manifest a wide range of clinical signs and symptoms, including fatigue, pruritus, night sweats, bone pain, and splenomegaly (Mesa et al., 2018; Mesa et al., 2021). Due to the increased blood volume and viscosity, patients are at an increased risk of both thrombotic and hemorrhagic events, which are the leading causes of morbidity and mortality in PV (Finazzi and Barbui, 2008; Marchioli et al., 2013; Griesshammer et al., 2019; Iurlo et al., 2020). Furthermore, PV has an inherent propensity of transformation to MF and acute myeloid leukemia (AML) over the long term (Cerquozzi and Tefferi, 2015; Stein et al., 2014). PV is associated with the risk of arterial and venous thrombosis and can be conventionally classified into two risk categories: high-risk (age >60 years or thrombosis history) and low-risk (age ≤60 years and no history of thrombosis) (Tefferi et al., 2021). The management of low-risk PV usually involves controlling hematocrit levels to reduce thrombotic risk, typically through phlebotomy and low-dose aspirin therapy, for patients who do not require cytoreductive therapy (Tefferi and Barbui, 2020). Hydroxyurea (HU) and polyethylene glycol-conjugated (PEGylated) interferon alpha (IFN-α) have often been used for cytoreduction in the management of high-risk patients (Bose and Verstovsek, 2019; Guglielmelli and Vannucchi, 2020). It has been shown that elevated WBC and platelet counts (>11×10^9^/L and >400×10^9^/L respectively) are correlated with increased thromboembolic (TE) risk (Bose and Verstovsek, 2019; Gerds et al., 2022). Therefore, the complete hematologic response (CHR) defined as a hematocrit <45% without phlebotomy, platelet count ≤400 × 10^9^/L, and WBC count <10 × 10^9^/L) is an important indicator for the efficacy of PV treatment and is used as an endpoint for the regulatory approvals (Gisslinger et al., 2020; Jin et al., 2023; Qin et al., 2024). Cytoreductive treatment has also been assessed in low-risk patients and has shown clinical benefits, including a reduced need of phlebotomy in the absence of thrombotic events and progression of leukocytosis or thrombocytosis (Barbui et al., 2023; Tefferi and Barbui, 2023).

IFN-α-based therapies have been shown to result in therapeutic cytoreduction and have been suggested to exhibit disease modifying potential in PV treatment (Masarova et al., 2017; Tashi et al., 2018; Abu-Zeinah et al., 2021; Kiladjian et al., 2022). IFN-α works together with its possible prototype IFN-β (Wittling et al., 2021), an important part of a network of tumor suppressors or their related proteins for the cell cycle-based anti-cancer surveillance (Qin et al., 1997; Kaynor et al., 2002; Qin, 2023a; Qin, 2024a). Furthermore, IFN-β/IFN-α induces tumor cell apoptosis if overexpressed, and can elicit natural killer cell and CD8^+^ lymphocytes-mediated anti-tumor activities (Qin et al., 1998; Qin et al., 2001; Brown et al., 2002; Zitvogel et al., 2015; Qin, 2023b).

Ropeginterferon alfa-2b (Ropeg) is a novel, site-selective, mono-PEGylated recombinant proline-IFN-α with a favorable pharmacokinetics (PK) profile (Huang et al., 2021; Huang et al., 2022; Miyachi et al., 2021). It showed robust CHRs, and safety in adult patients with PV across several clinical studies (Gisslinger et al., 2015; Gisslinger et al., 2020; Edahiro et al., 2022; Jin et al., 2023; Suo et al., 2024). Ropeg treatment has also been found to induce molecular responses by reducing *JAK2*V617F allele burden or variant allele frequency (VAF) under two different dosing regimens (Gisslinger et al., 2015; Gisslinger et al., 2020; Edahiro et al., 2022; Jin et al., 2023; Suo et al., 2024). It appears that Ropeg treatment at a higher starting dose and a simpler dose-titration regimen could lead to a greater molecular response (Jin et al., 2023; Suo et al., 2024). Ropeg is the only IFN-based therapy approved by the US Food and Drug Administration (FDA) for the treatment of patients with PV (FDA, 2019). The National Comprehensive Cancer Network (NCCN) has recently recommended Ropeg as the preferred cytoreductive treatment for patients with low-risk PV (NCCN. National Comprehensive Cancer Network, 2024). Understanding the ethnic sensitivity of Ropeg is vital for clinical practice. Ethnic sensitivity analysis addresses the genetic and physiological differences among populations that can influence drug metabolism and responses (ICH, 1998; Huang and Temple, 2008; Yasuda et al., 2008). This analysis is crucial for ensuring the global efficacy and safety of Ropeg, as it helps tailor treatment to diverse patient populations and aligns with the goals of personalized medicine (Bernal and Domenech Rodríguez, 2013; Vannucchi, 2017; Hong, 2023).

In this study, a comprehensive ethnic sensitivity analysis of Ropeg for the treatment of PV was performed. By comparing the global diagnostic and treatment criteria for PV and examining the PK, efficacy, and safety of Ropeg across different ethnic groups, we sought to understand its global applicability and effectiveness, contributing to the advancement of inclusive healthcare in managing PV.

## 2 Materials and methods

### 2.1 Clinical trial design and participants

This study encompassed a comprehensive analysis of data derived from phase I-III clinical trials of Ropeg, focusing on its application in the treatment of PV. The relevant clinical trial information is summarized in Table 1. The analysis was designed to assess the PK, efficacy, and safety of Ropeg across diverse ethnic groups with a particular emphasis on comparing the responses between Chinese and overseas populations of different ethnic backgrounds. The participant pool included adult patients diagnosed with PV, ensuring a broad representation of demographic variables such as age, sex, and ethnicity.

**TABLE 1:** Summary of clinical trials for ropeginterferon alfa-2b (Ropeg).

Study	Study design	Population	Sample size	Dose
Clinical studies in healthy subjects				
A09-102 (Huang et al., 2021) (2009–2010)	Single-center, double-blind, randomized controlled single-dose escalation	Caucasian and Black adult males	48 subjects	12 subjects received peginterferon alfa-2a (180 μg); 36 subjects received ropeg (24 μg, 48 μg, 90 μg, 180 μg, 225 μg, or 270 μg)
A17-101 (Huang et al., 2021) (2019)	Randomized, open-label, positive control, single center, dose-escalation	Chinese	40 subjects (10 subjects in each group)	Peginterferon alfa-2a: 180 μg Ropeg: 90 μg, 180 μg, or 270 μg
Clinical studies in patients with PV				
PEGINVERA (Gisslinger et al., 2015) (2010–2018)	Open-label, prospective, multicenter, phase I/II dose escalation	Caucasian, Asian	51 patients	Stage 1: Ropeg (50 μg, 100 μg, 150 μg, 225 μg, 300 μg, 360 μg, 450 μg, or 540 μg) Stage 2: Ropeg (100 μg, 150 μg, 225 μg, 300 μg, 400 μg, and 450 μg)
PROUD-PV (Gisslinger et al., 2020) (2013–2016)	Randomized, open-label, multicenter, controlled, parallel arm, phase III	Caucasian	254 patients 127 in each group; Ropeg vs. hydroxyurea (HU)	Ropeg: Starting dose at 100 μg (or 50 μg for patients receiving HU) Intrapatient Ropeg dose titrations by 50 μg every 2 weeks until hematological parameters reaching steady state Maximum recommended single dose was 500 μg every 2 weeks
CONTINUATION-PV (Gisslinger et al., 2020) (2014–2021)	Open-label, multicenter, phase IIIb	Caucasian patients who previously participated in PROUD-PV Study	171 patients 95 in Ropeg arm 76 in control arm	PROUD-PV maintenance dose
PEN-PV (EMA, 2018) (2015)	Open-label, single-arm, phase III study using a pre-filled pen	Caucasian	36 patients who completed PROUD-PV (*n* = 5) or participated in CONTINUATION-PV study (*n* = 31)	PROUD-PV maintenance dose
A19-201 (Edahiro et al., 2022) (2019–2021)	Phase II single-arm	Japanese patients with PV; unsuitable with the current standard of treatment	29 patients	Same as PROUD-PV
^a^A20-202 (Jin et al., 2023) (2020–2023)	Phase II single-arm	Chinese PV patients with HU intolerance	49 patients	The 250-350–500 µg dosing regimen. Week 0: 250 µg Week 2: 350 µg Week 4: 500 µg Maintenance dose of 500 µg if tolerated; Dose can be adjusted according to tolerability

^a^ E-R analysis was based on treatment data at 24 weeks.

Data collection encompassed a wide array of parameters, including patient demographics, specific Ropeg dosing regimens, PK measurements, efficacy outcomes, and adverse events (AEs). The PK data focused on capturing the temporal profile of Ropeg plasma concentrations after administration, providing a dataset for subsequent analyses.

### 2.2 PK analysis

A population PK (PopPK) model was used to analyze the PK behavior of Ropeg. This model is instrumental in identifying and quantifying the effects of various covariates, including ethnic background, on drug metabolism and distribution. The PopPK model was calibrated and validated using PK data to ensure robustness and reliability in assessing ethnic variations.

### 2.3 Exposure-response analyses

The efficacy of Ropeg was evaluated against the established clinical response criteria for PV. These criteria encompassed a range of hematological and clinical parameters, providing a comprehensive view of the therapeutic impact. The safety assessment focused on the incidence, severity, and pattern of AEs across different ethnic groups. The dual focus on efficacy and safety is pivotal for providing a balanced view of the therapeutic profile of Ropeg.

Logistic regression models are a key component of this framework, enabling the examination of the relationship between drug exposure and clinical response. These models were complemented by additional statistical tests and comparative assessments to elucidate any significant differences in PK and safety profiles across ethnic groups. Statistical significance was determined using predetermined alpha levels and confidence intervals were used to gauge the precision of the estimates.

### 2.4 Ethical considerations

All clinical trials included in the analyses adhered to ethical standards, in line with the principles of the 1,964 Helsinki Declaration and its subsequent amendments. Informed consent was obtained from all participants involved in the studies. Ethical rigor ensured the integrity and soundness of the study.

## 3 Results

### 3.1 Diagnosis, treatment, and efficacy assessment in PV

For the diagnosis of PV, the *Chinese Guidelines for the Diagnosis and Treatment of Polycythemia Vera* (Leukemia and Lymphoma Group, Chinese Society of Hematology, Chinese Medical Association, 2022) recommend adhering to the diagnostic criteria set forth by the World Health Organization (WHO) (Barbui et al., 2018). To evaluate the efficacy of PV treatment in Chinese patients, Chinese guidelines recommend referring to the revised criteria of the European Leukemia Net (ELN) and the International Working Group for Myeloproliferative Neoplasms Research and Treatment (IWG-MRT) (Barosi et al., 2013). This dual-reference framework integrates international standards into the Chinese clinical context, ensuring a rigorous and globally informed approach to both diagnosis and response assessment in PV management.

The clinical phenotypes or manifestations of PV can be heterogeneous. The use of phlebotomy, cytoreductive agents such as HU, IFN-α-based treatment, and second-line therapy with the JAK inhibitor ruxolitinib can also vary depending on individual physicians providing the treatment (Palumbo et al., 2023). In addition, clonal heterogeneity with *JAK2* mutations and heterogeneous gene expression have been observed in patients with PV (Li et al., 2008; Spivak et al., 2014). However, the overall treatment principles, particularly regarding the use of IFN-α-based therapies, were not observed to have major discrepancies among different ethnic groups. IFN-α-based therapies have been shown to exert disease-modifying effect by prolonging progression-free survival, event-free survival, and potentially, overall survival in patients with PV (Abu-Zeinah et al., 2021; Gisslinger et al., 2023). The Chinese Guidelines for the Diagnosis and Treatment of Polycythemia Vera, NCCN guidelines, and Austrian Treatment Recommendations for Polycythemia Vera 2018 (Leukemia and Lymphoma Group, Chinese Society of Hematology, Chinese Medical Association, 2022; NCCN. National Comprehensive Cancer Network, 2024; Burgstaller et al., 2018) all endorse the IFN-α-based treatment as a first-line therapy for PV. This consensus highlights a global alignment in the therapeutic approach for PV treatment regarding the use of disease modifying therapies.

Therefore, the clinical standards for the diagnosis and treatment of PV are consistent across Chinese and overseas populations with different ethnic backgrounds. In clinical studies using Ropeg for PV treatment, similar diagnostic and efficacy evaluation criteria were applied. There were no significant ethnic differences in PV pathophysiology, diagnosis, or efficacy evaluations between Chinese and overseas populations with different ethnic backgrounds.

### 3.2 PK profiles in Chinese and Caucasian subjects

Data from phase I clinical studies A09-102 (Huang et al., 2021) and A17-101 (Huang et al., 2022) conducted on healthy subjects were utilized to construct the PopPK model PM-202002 (Zhu et al., 2021). A09-102 was conducted mostly in Caucasian subjects, while A17-101 was conducted in Chinese subjects. This model aimed to analyze the PK variance of Ropeg in healthy Chinese and Caucasian adult subjects. The analysis incorporated data from 57 participants (894 measurements). Covariate inclusion in the analysis was performed, highlighting a demographic composition of 52.63% Caucasians, and 47.37% Chinese. Age in the two ethnic groups was comparable (32.3 ± 6.9 vs. 31.6 ± 6.19), whereas the body weight was 14% lower in the Chinese population than the Caucasian population (68.1 ± 10 vs. 79.4 ± 9.41 kg) (Zhu et al., 2021). In the groups receiving identical doses (90 μg, 180 μg, or 270 μg), the plasma concentration-time profiles for subjects included in the analysis were demonstrated. Notably, there was a high degree of similarity between the concentration-time curves of the Chinese and Caucasian populations, indicating comparable PK behaviors between these groups. Furthermore, PopPK analysis identified ethnicity as a categorical variable representing Chinese and Caucasian populations in the covariate modeling analysis. Ethnicity showed no significant impact on the typical values for clearance (CL/F), volume of distribution (Vc/F), and absorption rate constant (k_a_) (Zhu et al., 2021), confirming the absence of a substantial difference in PK parameters between the Chinese and Caucasian populations.

Data from three clinical studies, A09-102, PEGINVERA, and PROUD-PV, were utilized in PopPK model 0158-1 (FDA, 2020). The PEGINVERA was conducted mostly in Caucasian patients with PV, whereas the PROUD-PV was conducted in Caucasian patients with PV. The model was designed to describe the plasma concentration-time profile of Ropeg following subcutaneous (SC) administration.

A comparative analysis of the parameter estimations from the two PopPK studies PM-202002 and 0158-1 is further summarized in Table 2. The results indicate that the CL/F and Vc/F were similar between the studies with the population typical values of Vc/F being 2.32 and 2.56 L, a difference of not higher than 10%; and those for CL/F being 0.85 and 1.12 L/day at 76.7 kg, a difference of not higher than 25%. This similarity suggests a comparable PK behavior between Chinese and Caucasian subjects. Furthermore, both PopPK analyses demonstrated that a non-linear increase in clearance was correlated with an increase in body weight. The findings from the PopPK analyses suggest that the PK behavior of Ropeg is similar between Chinese and Caucasian subjects, indicating that no significant ethnic differences exist in PK.

**TABLE 2:** Comparison of parameter estimates from two PopPK analyses.

PK parameters	PM-202002-1	0158-1	Ratio of population typical value
CL/F (L/day)	0.778 × (WT/70)^0.927^	1.12 × (BSA/1.92)^1.15^ + 0.000164 × (JAK2-35.3)	0.76^a^
V_c_/F (L)	2.32	2.56 × (1 + 0.892 × PV)	0.91
k_a_ (1/day)	0.14	0.120 × (age/54.3)^−0.655^	1.17
t_lag_ (h)	0.426	0.658	0.65
R_tot,0_ (ng/mL)	0.111	0.322	0.34
k_int_ (1/h)	0.0788 FIX	0.0333	2.36
k_deg_ (1/h)	0.544	0.362	1.50
K_D_ (ng/mL)	0.142 FIX	0.181	0.78

^a^ The typical population value for CL/F (L/d) was calculated based on a median body weight of 76.7 kg (FDA, 2020). Abbreviations: CL/F, apparent clearance; k_a_, absorption rate constant; k_int_, first-order elimination rate constant of the complex; k_deg_, first order rate constant of target receptor degradation; K_D_, binding dissociation constant; R_tot,0_, initial maximum target receptor binding capacity; t_lag_, absorption lag time; V_c_/F, apparent central volume distribution.

### 3.3 Exposure-response analyses in Chinese and Caucasian patients

#### 3.3.1 Exposure-efficacy analysis in Chinese and Caucasian patients

Data from a phase II clinical study, A20-202, in Chinese patients with PV were utilized for an exposure-efficacy analysis (Qin et al., 2024), leading to the development of a logistic regression model correlating exposure (C_avg,0-24w_) with efficacy, i.e., CHR. The analysis included 48 patients with PV, after excluding one patient due to early withdrawal from the study. To calculate the mean plasma concentrations over 24 weeks (C_avg_,_0-24w_), the exposures were simulated based on the actual doses administered to each patient. The exposure levels categorized by quartiles are listed in Supplementary Table S1. They were then compared with exposure over 24 weeks in simulated patients in the PROUD-PV study (Gisslinger et al., 2020). Patients in A20-202 were administered Ropeg at a higher starting-dose regimen with faster dose-titrations (i.e. the 250-350–500 µg schema), while patients in PROUD-PV received the approved slow dose-titration schema as shown in Table 1.

**FIGURE 1:**
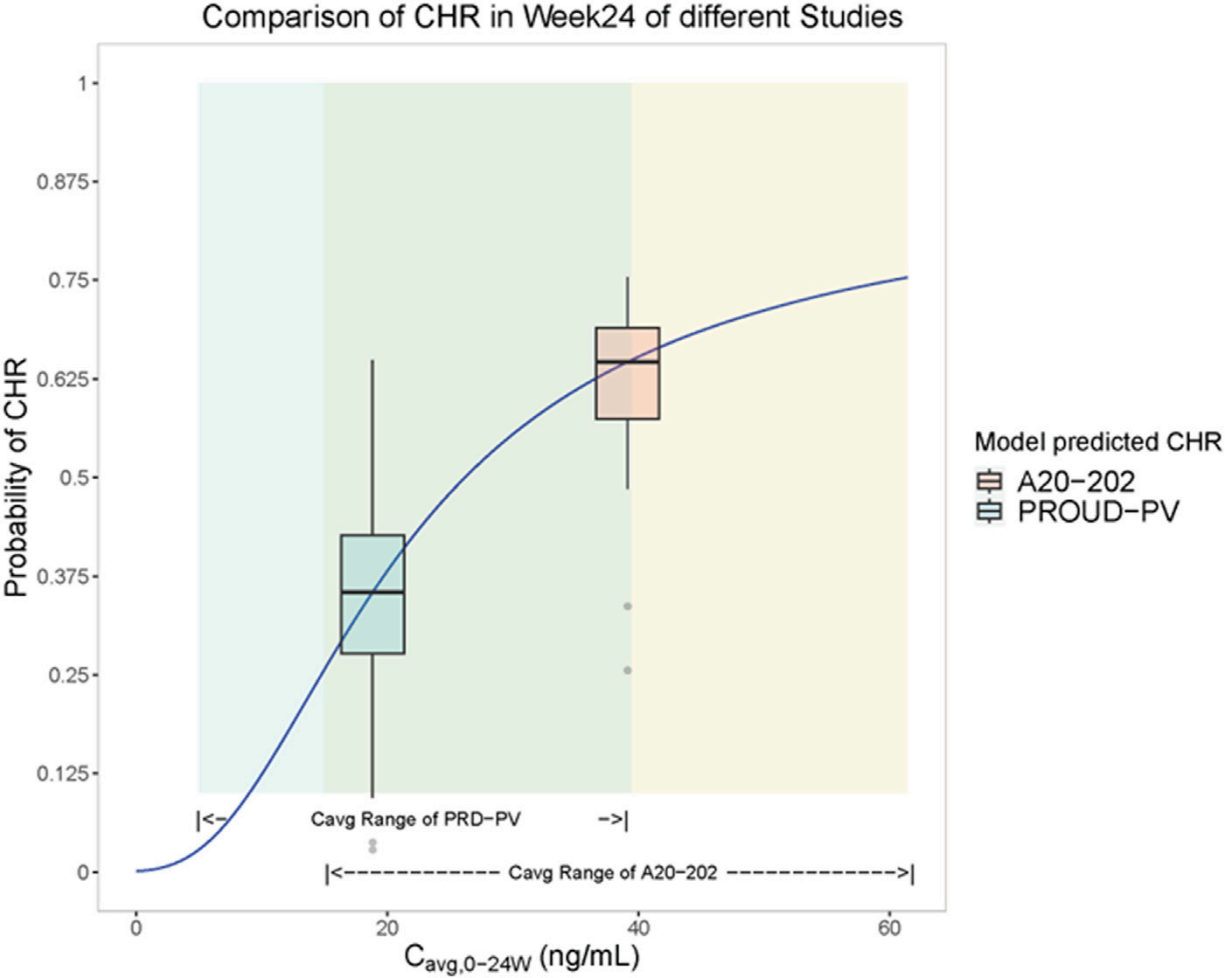
Visualization of the model simulation for the exposure-efficacy relationship: C_avg,0-24w_ - complete hematologic response (CHR) rate logistic regression model analysis (Solid blue lines are fitted to C_avg, 0-24w_-CHR rate logistic regression models). Yellow shading represents exposure (C_avg, 0-24w_) range over 24 weeks for subjects in A20-202 (*n* = 48) and green shading represents simulated exposure (C_avg, 0-24w_) range over 24 weeks of PROUD-PV based on the available population typical values of PK parameters (FDA, 2020). Yellow box plots represent the predicted range of CHR rates in A20-202 study, the middle horizontal black line of the yellow box represents the median CHR rate over the exposure range in A20-202, and the vertical line of the yellow box corresponds to the abscissa of the median A20-202 trial exposure. The green box plot represents the predicted range of CHR rates in the PROUD-PV study, the middle horizontal black line of the green box represents the median CHR rate over the exposure range in PROUD-PV conducted in Europe, and the horizontal line corresponds to the median of PROUD-PV trial exposure.

A visualization of the exposure-efficacy relationship model is illustrated in Figure 1. Based on the model simulation, patients in A20-202 were estimated to achieve a median CHR rate of approximately 63% at Week 24. In contrast, PROUD-PV, in which Caucasian patients treated with a slow dose-titration regimen (indicated in green), was estimated to have a CHR rate of approximately 35% in the same interval. This is consistent with the clinically observed CHR rates of 61.2% at Week 24 for A20-202 and 27% in PROUD-PV, respectively (Jin et al., 2023; Gisslinger et al., 2020). The fact that the model-simulated CHR rates were similar to the clinically realistic rates indicates that there is a similar exposure-response relationship between Chinese and Caucasian populations. The median predicted CHR rate was notably higher in A20-202 than in PROUD-PV because of the higher exposure resulting from the higher starting-dose regimen with faster dose-titrations used in A20-202.

*JAK2*V617F is a driver mutation that is present in most PV cases (Baxter et al., 2005; Tefferi et al., 2021). The reduction of *JAK2*V617 allele burden has emerged as an important indicator of treatment effect in PV as it appears to be associated with decreased risk of thrombosis, progression-free survival, and event-free survival (Stein et al., 2014; Harrison et al., 2023; Gisslinger et al., 2023; Moliterno et al., 2023). Ropeg treatment has been shown to reduce the *JAK2*V617F allele burden in patients with PV across multiple studies conducted in Europe, Japan, and China (Gisslinger et al., 2015; Gisslinger et al., 2020; Edahiro et al., 2022; Jin et al., 2023; Suo et al., 2024). Consistent with the dose-exposure-CHR data, greater reduction of the *JAK2*V617F allele burden was also observed in patients treated with Ropeg at the higher starting dose regimen and the burden reduction increased with increasing exposure (Jin et al., 2023; Suo et al., 2024; Qin et al., 2024). Ropeg represents a new-generation IFN-α-based therapy. IFN-α exerts its biological activities by binding to its the receptors IFNAR1 and 2 located on cell membranes. The mechanism of action of Ropeg in PV treatment is likely to be at least due to the activation of downstream tumor suppressors or their related proteins, and the inhibition of the relevant proto-oncogenes (Qin, 2024b). Therefore, the mechanism of action and effect of Ropeg on the *JAK2*V617F allele burden do not appear to differ with regards to ethnic variations.

#### 3.3.2 Exposure-safety analysis in Chinese and Caucasian patients with PV

To analyze exposure-safety correlations, a logistic regression model was developed using data from the phase II clinical study A20-202 up to Week 24 in Chinese patients with PV (Qin et al., 2024). This study had exposure data categorized into different dose phases including 250 μg, 350 μg, and 500 μg, based on the actual doses administered. The average concentration of each phase (C_avg,250 μg_, C_avg,350 μg_, and C_avg,500 μg_) was calculated. The analyzed safety indices included drug-related AEs, Grade 3 or higher treatment-related AEs, and AEs with higher incidence rates. These include increases in gamma-glutamyl transferase (GGT), alanine aminotransferase (ALT), aspartate aminotransferase (AST), and asthenia and decreases in WBC and neutrophil counts (Qin al., 2024). No apparent correlation between the high frequency AEs and exposure to Ropeg was observed, except that increases in ALT and AST appeared to be associated with the average Ropeg exposure in the 500 µg phase. Other safety indices, including increased GGT, WBC, and neutrophil count decrease, and asthenia were not affected by the exposure levels, as shown in Supplementary Figures S1-S8. The ALT and AST levels did not increase with significant increases in bilirubin, clinical symptoms, or signs, such as jaundice (Jin et al., 2023; Suo et al., 2024). These results suggest that the safety risks associated with Ropeg treatment are acceptable.

Next, an exposure-safety analysis was performed using data from four clinical studies involving European patients with PV: PEGINVERA, PROUD-PV, CONTINUATION-PV, and PEN-PV (Table 1). This analysis included 178 patients who had been treated with a slow-dose titration regimen of Ropeg. The safety indices assessed included drug-related AEs, drug-related AEs of Grade 3 and higher, serious adverse events (SAEs), and drug-related SAEs. The analysis concluded that the exposure-safety relationships were acceptable, and the side effects were tolerated by most patients.

The exposure-safety analysis results in Chinese patients with PV align with those observed in Caucasian patient populations, indicating a similar safety profile for Ropeg in both Chinese and Caucasian individuals. These results suggest that the risk of AEs related to transaminases has increased. However, most cases were mild or moderate and did not require treatment discontinuation, reflecting an acceptable safety profile. The results indicated that Ropeg were consistently well-tolerated by the two ethnic groups.

## 4 Discussion

The comprehensive review and analyses in this study support the global applicability of Ropeg and highlight the effectiveness as a preferred first line cytoreductive intervention for patients with low- or high-risk PV among different ethnicities. The convergence of the diagnostic and efficacy evaluation criteria, in line with the WHO and ELN criteria, further validated the uniformity of diagnostic and response assessment practices.

Multiple PopPK analyses were conducted using data from healthy Chinese and Caucasian individuals and patient populations. The results showed that the drug concentration-time profiles in the different populations were similar, suggesting comparable PK behaviors. Ethnicity showed no significant impact on the PK parameters, confirming the absence of substantial differences in PK characteristics between the Chinese and Caucasian populations. The slight difference in the PK of Ropeg between Caucasian and Chinese subjects might be caused by differences in body weight. This is consistent with our previous data that PK exposure was observed to be higher in Japanese than in Caucasian subjects, largely due to differences in the body weight, without exerting an overt effect on pharmacodynamic parameters, safety, and tolerability (Miyachi et al., 2021). Our model simulation analysis further suggested that the Chinese and Caucasian populations exhibited a similar dose-effect relationship, and the exposure-response profiles for Ropeg were comparable, suggesting a uniform therapeutic effect irrespective of ethnic variations. Similarly, the exposure-safety analysis indicated that Ropeg is well-tolerated among different ethnicities. The increased ALT and AST levels observed in Chinese patients with PV were most likely due to the use of a higher starting-dose regimen with simpler and faster dose titrations. However, transaminase increases are reversible and do not appear to be associated with significant increases in bilirubin levels or clinical signs and symptoms (Jin et al., 2023; Suo et al., 2024). Therefore, the positive benefit-over-risk balance with Ropeg treatment in patients with PV did not appear to change with a higher starting-dose regimen as a treatment option.

The results revealed no significant ethnic variation in the PK, efficacy, or safety profiles of Ropeg, which encompassed multiple clinical trials and PK analyses across ethnic cohorts, including Chinese and Caucasian populations. These findings are pivotal considering the slight PK differences that could be attributed to variations in body weight rather than ethnicity. The exposure-efficacy and exposure-safety analyses demonstrated analogous dose-effect relationships and safety profiles across different ethnic groups. This consistency is helpful in confirming the universal applicability of Ropeg and supporting its use in diverse populations. Finally, although we did not observe overt differences in pharmacodynamic parameters, safety and tolerability due to higher PK exposure in subjects with low body weight (Miyachi et al., 2021), it is reasonable to take caution when titrating the dose, with close monitoring in patients with very low body weights to avoid unwanted side effects caused by a higher PK exposure.

Our analyses had some limitations. PV is known to be heterogeneously associated with unique gene mutations or patterns regarding *JAK2*V617F, *JAK2* exon 12 mutations and/or others (Vannucchi et al., 2007; Patnaik and Tefferi, 2009; Passamonti et al., 2011; Kanduła et al., 2023). Variations in non-driver mutations, including *ASXL1*, *SRSF2*, and *IDH2*, and differential blood counts can also lead to phenotypic heterogeneity, such as the risk of thrombosis and disease progression (Landolfi et al., 2007; Gangat et al., 2007; De Stefano et al., 2010; Tefferi et al., 2016; Gerds et al., 2022). Although most of the patients in the analyzed studies were known to have *JAK2*V617F, there is a lack of sufficient data or thorough analyses regarding the effect of Ropeg in patients with other *JAK2* mutations, or with *JAK2*V617F but carrying different non-driver mutations, or with other relevant gene variations. There is also a lack of integrated analyses across various clinical studies to assess the effect of Ropeg in patients with different levels of leukocytes and platelets. However, Ropeg treatment as an IFN-α-based therapy can induce the type 1 IFN signaling leading to the activation of downstream, cell cycle- and senescence-regulatory tumor suppressor gene products or related proteins, which could impose gene and epigenetic regulations to inhibit the neoplastic cells that drive PV (Qin, 2023a; Qin, 2024b). Ropeg may be efficacious in these patients, although subtle differences in PK, efficacy, and safety might be present. Our study also did not analyze the data of patients with non-PV erythrocytosis, such as those with congenital polycythemia, who have an increased risk of thrombosis due to other mutations (Gordeuk et al., 2004; Tomasic et al., 2013; Gangat et al., 2021). However, Ropeg treatment was recently shown to induce remission in a patient carrying *JAK2* ^R715T^ from a germline origin (Song et al., 2024), supporting the broad therapeutic effect of Ropeg. Finally, our study did not include an overall pharmacoeconomic analysis to assess the global affordability of Ropeg as a frontline treatment of PV. Ropeg was previously found to be a cost-effective treatment option for a broad range of patients with PV in the US (Gerds et al., 2023). Further analyses of pharmacoeconomics in other countries or regions will be needed to evaluate the clinical application of Ropeg in diverse patient populations.

## 5 Conclusion

Our results support the use of Ropeg as an effective and tolerable first-line treatment for PV regardless of ethnic variations. The findings have implications for the clinical management of PV in patients of various ethnic backgrounds receiving Ropeg treatment. Future research may extend to explore any differences in treatment responses or side effects in patients with other *JAK2* or non-*JAK2* driver mutations besides *JAK2*V617F, differential non-driver mutations, or other relevant genetic variations to enhance personalized medical approaches in the PV treatment.

## Data Availability

The original contributions presented in the study are included in the article/Supplementary Material, further inquiries can be directed to the corresponding author.
