# Supplementary material for: Ethnic sensitivity analyses of pharmacokinetics, efficacy and safety in polycythemia vera treatment with ropeginterferon alfa-2b
Source: Front Pharmacol. 2024 Sep 24;15:1455979. doi: 10.3389/fphar.2024.1455979 (PMC11463156; doi:10.3389/fphar.2024.1455979)
Supplement: Supplementary file 1 [file DataSheet1.docx]

*Supplementary Materials*

# Ethnic Sensitivity Analyses of Pharmacokinetics, Efficacy and Safety in Polycythemia Vera Treatment with Ropeginterferon alfa-2b

Albert Qin, MD PhD^1*^, Daoxiang Wu, MD^2^, Jason Liao PhD^1^, Shuping Xie, PhD^3^, Haoqi Chen^3^, Yucheng Gao^3^, Jie Cui^3^, Xia Su PhD^3^, Narihisa Miyachi PhD^4^, Toshiaki Sato, MD PhD^4^, Yaning Li^2^, Jingjing Zhang^2^, Weihong Shen^2^, Wei Wang, MD^2^

*^1^ Medical Research & Clinical Operations, PharmaEssentia Corporation, Taipei, Taiwan, Republic of China*

*^2^* *PharmaEssentia Biotech (Beijing) Limited, Beijing, China*

*^3^* *Pharmaron Clinical Services Co., Ltd, Chengdu, China*

*^4^ PharmaEssentia Japan KK, Tokyo, Japan*

**Table S1. Summary of Exposure by Interquartile Range in Study A20-202**

|  | Q1 | Q2 | Q3 | Q4 | Total (*n* = 48) |
| --- | --- | --- | --- | --- | --- |
| **C_avg,0-24w_ (ng/mL)** | | | | | |
| Mean (SD) | 26.77 (5.20) | 35.41 (2.58) | 41.76 (2.06) | 53.23 (5.39) | 39.29 (10.52) |
| Median [min, max] | 29.27  [14.94,30.95] | 34.91  [31.75,38.94] | 41.44  [39.3,45.53] | 51.93  [46.13,61.41] | 39.12  [14.94,61.41] |


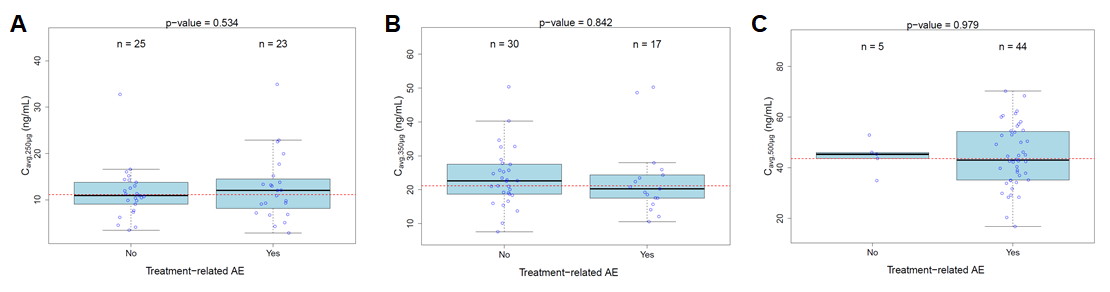


**Figure S1. Plot of relationship between exposure and occurrence of adverse events. Relationship between (A) C_avg, 250 μg_, (B) C_avg, 350 μg_, and (C) C_avg, 500 μg_ and the occurrence of drug-related TEAEs following ropeg administration, respectively. Blue dots represent individual patient exposure values, calculated via simulations from the population pharmacokinetic model. The box plots indicate the distribution of exposure in patients, with the middle horizontal black line denoting the median. The box's lower and upper bounds represent the 25th and 75th percentiles, while the horizontal lines extending from each box indicate 1.5 times the interquartile range (IQR). The 'Yes' and 'No' labels signify the occurrence or absence of adverse events. The red dashed horizontal line across the plot indicates the overall median exposure for both 'Yes' and 'No' groups.**


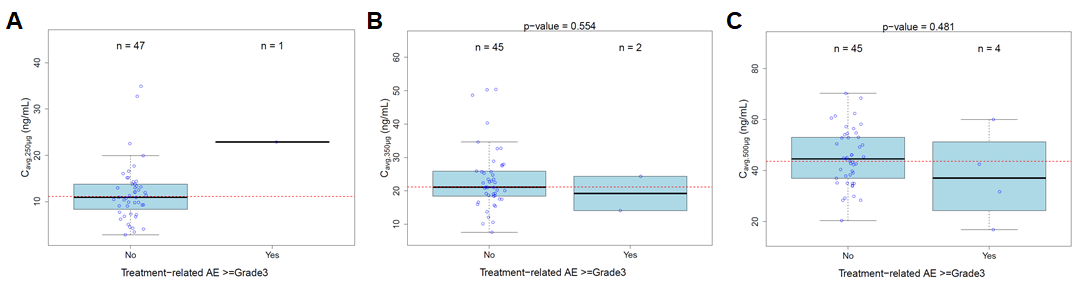


**Figure S2. Analysis of Severe Drug-Related TEAEs and ropeg Exposure Levels. The figure demonstrates the association between various average exposure levels of ropeg (C_avg_ at 250 μg (A), 350 μg (B), and 500 μg(C)) and the frequency of grade 3 or higher drug-related treatment-emergent adverse events (TEAEs). Blue dots show individual patient exposure, calculated from population pharmacokinetic model simulations. Each box plot illustrates the exposure distribution among patients, with the median marked by a middle horizontal black line. The 25th and 75th percentiles are indicated by the lower and upper ends of the box, respectively. The whiskers extend to 1.5 times the interquartile range (IQR) from the box. Labels 'Yes' and 'No' indicate the presence or absence of severe TEAEs. The red dashed horizontal line across the plots represents the overall median exposure for both 'Yes' and 'No' groups.**


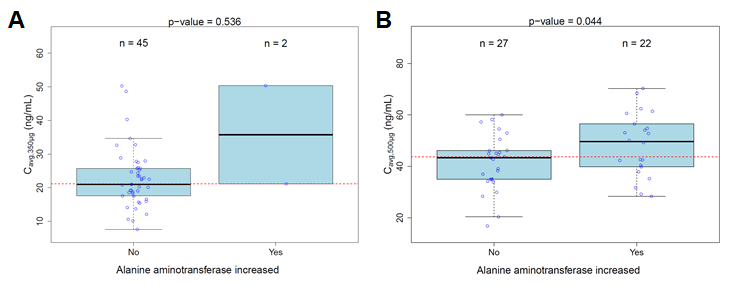


**Figure S3. Correlation Between ropeg Exposure Levels and Increased Alanine Aminotransferase This figure presents the relationship between two dosage levels of ropeg (C_avg_ at 350 μg (A) and 500 μg (B)) and the incidence of increased alanine aminotransferase (ALT) post-administration. Note that no such adverse events were reported during the 250 µg dose period. Blue dots denote individual patient exposure values, determined via simulations from the population pharmacokinetic model. Each box plot displays the exposure distribution among patients, with the median represented by the middle horizontal black line. The box's lower and upper boundaries indicate the 25th and 75th percentiles, while the horizontal lines extending from the box show 1.5 times the interquartile range (IQR). The labels 'Yes' and 'No' signify the occurrence or absence of increased ALT, and the red dashed horizontal line marks the overall median exposure for these two groups.**

**
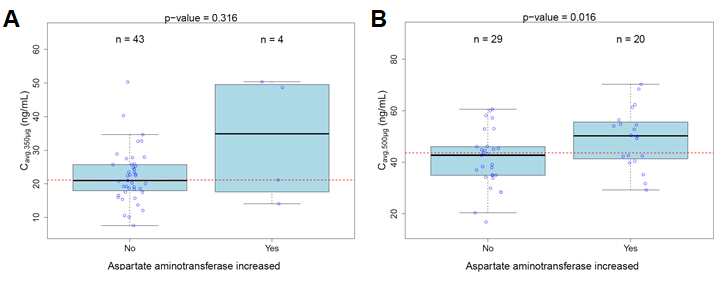
**

**Figure S4. Analysis of Increased Aspartate Aminotransferase (AST) in Relation to ropeg Exposure at 350μg and 500μg Doses. This figure depicts the correlation between two specific dosage levels of ropeg (C_avg_ at 350 μg (A) and 500 μg (B)) and the incidence of increased AST following administration. The blue dots represent individual patient exposure values, calculated using simulations from the population pharmacokinetic model. In each box plot, the median exposure level is indicated by a middle horizontal black line, while the lower and upper ends of the box mark the 25th and 75th percentiles, respectively. The whiskers, or horizontal lines at both ends of the box, extend to 1.5 times the interquartile range (IQR). The labels 'Yes' and 'No' categorize patients based on the occurrence or absence of increased AST, respectively. The red dashed horizontal line across the plots highlights the overall median exposure for both the 'Yes' and 'No' groups.**

**
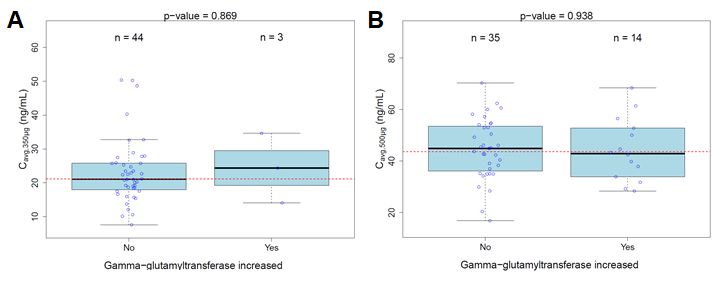
**

**Figure S5. Relationship Between Higher Doses of ropeg and Increased Gamma-Glutamyltransferase (GGT) Levels. This figure shows the correlation between average concentrations of ropeg (C_avg_ at 350 μg (A) and 500 μg (B)) and the incidence of increased GGT levels following drug administration. Notably, no cases of increased GGT were reported at the 250 μg dose. Blue dots represent individual patient exposures, calculated from simulations using the population pharmacokinetic model. Each box plot displays the distribution of exposure levels: the median is shown by the middle horizontal black line, and the 25th and 75th percentiles are indicated by the lower and upper ends of the box, respectively. Horizontal lines at both ends of each box extend to 1.5 times the interquartile range (IQR). The labels 'Yes' and 'No' classify occurrences of increased GGT. Additionally, the red dashed horizontal line across the plots represents the overall median exposure for both groups.**

**
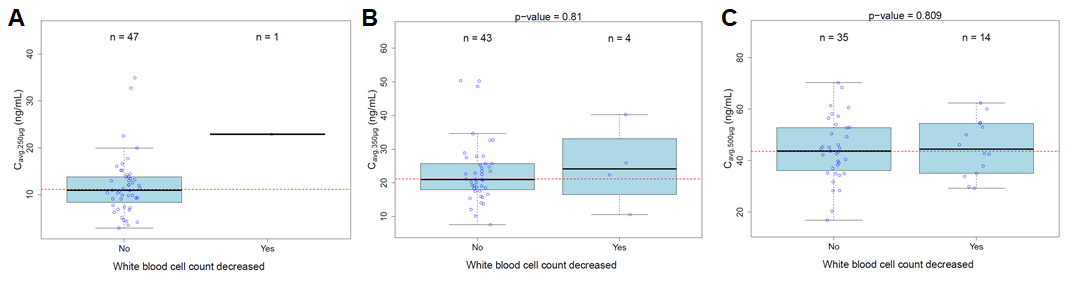
**

**Figure S6. Correlation Between ropeg Exposure Levels and White Blood Cell (WBC) Changes . This figure illustrates the relationship between different average concentrations of ropeg (C_avg_ at 250 μg (A), 350 μg (B), and 500 μg (C)) and the occurrence of changes in white blood cell count post-administration. Blue dots represent individual patient exposure levels, derived from simulations based on the population pharmacokinetic model. The box plots depict the distribution of these exposure levels: the median is indicated by the middle horizontal black line, while the 25th and 75th percentiles are marked by the lower and upper ends of the box. The horizontal lines extending from each box represent 1.5 times the interquartile range (IQR). The labels 'Yes' and 'No' classify patients based on the presence or absence of WBC changes. The red dashed horizontal line across the plots signifies the overall median exposure for both 'Yes' and 'No' groups.**

**
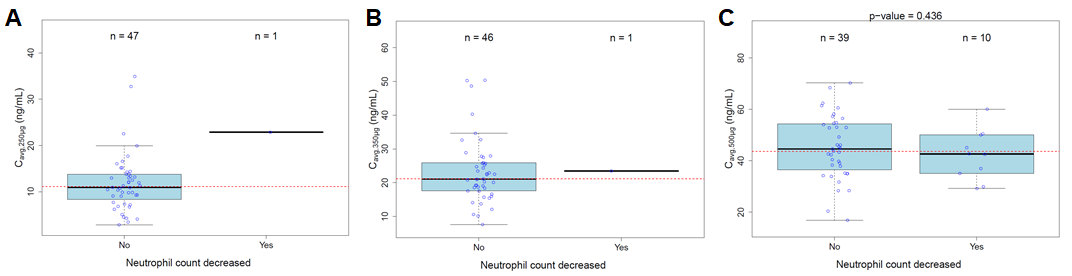
**

**Figure S7. Association Between ropeg Exposure and Neutrophil Count Decrease. This figure analyzes the relationship between three average concentration levels of ropeg (C_avg_ at 250 μg (A), 350 μg (B), and 500 μg (C)) and the incidence of neutrophil count decrease following administration. Individual patient exposures, represented by blue dots, are calculated from the population pharmacokinetic model simulations. The box plots provide a statistical summary of these exposures: the median is shown by the middle horizontal black line, and the 25th and 75th percentiles are indicated by the lower and upper ends of the box, respectively. The whiskers, or horizontal lines at each end of the box, extend to 1.5 times the interquartile range (IQR). The 'Yes' and 'No' labels indicate the occurrence or absence of a neutrophil count decrease. Additionally, the red dashed horizontal line across the figure denotes the overall median exposure for both 'Yes' and 'No' groups.**

**
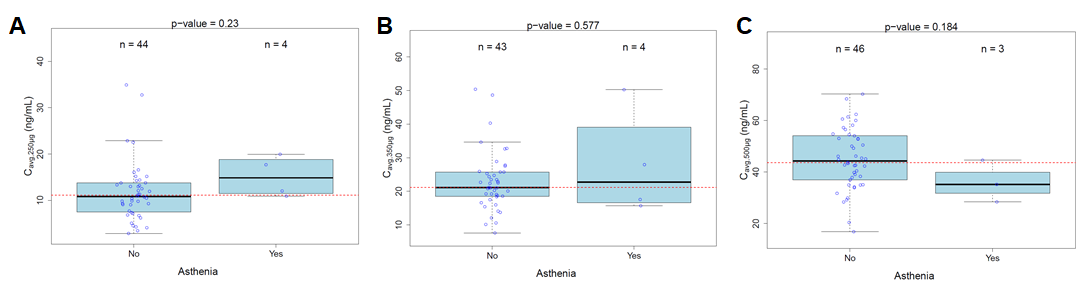
**

**Figure S8: Correlation Between ropeg Doses and Asthenia Incidence. This figure illustrates the relationship between varying doses of ropeg (C_avg_ at 250 μg (A), 350 μg (B), and 500 μg (C)) and the occurrence of asthenia in patients. The blue dots show individual patient exposure levels, determined through simulations based on the population pharmacokinetic model. The box plots represent the statistical distribution of these exposure levels: the median is marked by a middle horizontal black line, while the 25th and 75th percentiles are indicated at the lower and upper ends of the box. The horizontal lines extending from each end of the box correspond to 1.5 times the interquartile range (IQR). The 'Yes' and 'No' labels categorize patients based on the presence or absence of asthenia, respectively. Furthermore, the red dashed horizontal line across the figure indicates the overall median exposure for both 'Yes' and 'No' groups.**
